# Supplementary figures and images for: A Snapshot of the UK Blood Donor Plasma Virome: A Retrospective Cross‐Sectional Cohort Study
Source: J Med Virol. 2026 Jul 27;98(8):e71081. doi: 10.1002/jmv.71081 (PMC13408318; doi:10.1002/jmv.71081)

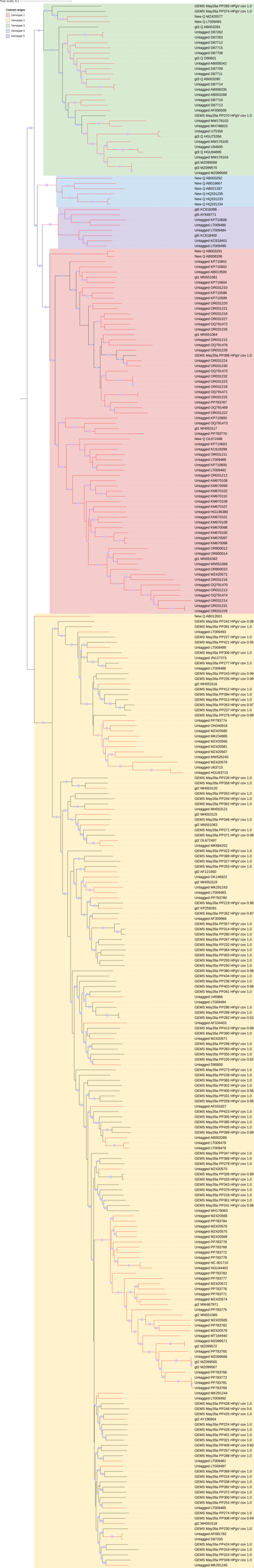

Supplement: Supplementary file 4 — Supporting File 4 [file JMV-98-e71081-s004.pdf]
